# A novel praziquantel solid lipid nanoparticle formulation shows enhanced bioavailability and antischistosomal efficacy against murine *S. mansoni* infection

**DOI:** 10.1186/s13071-019-3563-z

**Published:** 2019-06-17

**Authors:** Amr Radwan, Naglaa M. El-Lakkany, Samia William, Gina S. El-Feky, Muhammad Y. Al-Shorbagy, Samira Saleh, Sanaa Botros

**Affiliations:** 1 Research Department, Academy of Scientific Research and Technology, Cairo, Egypt; 2 Pharmacology Department, Theodor Bilharz Research Institute, Imbaba, Giza, Egypt; 3 Parasitology Department, Theodor Bilharz Research Institute, Imbaba, Giza, Egypt; 4 Pharmaceutical Technology Department, National Research Center, Giza, Egypt; 5 Department of Pharmacology & Toxicology, Faculty of Pharmacy, Cairo University, Giza, Egypt; 6 School of Pharmacy, Newgiza University, Giza, Egypt

**Keywords:** Bioavailability, Efficacy, Mice, Praziquantel, *Schistosoma mansoni*, Solid lipid nanoparticles

## Abstract

**Background:**

Schistosomiasis is responsible for a considerable global disease burden. This work aimed to improve the therapeutic outcome of the only available antischistosomal drug worldwide, praziquantel (PZQ), by incorporating it into a novel carrier, “solid lipid nanoparticles (SLNs)”, to enhance its solubility, bioavailability and efficacy. A simple, cost-effective method was used to prepare SLN-PZQ.

**Results:**

Compared to market PZQ (M-PZQ), SLN-PZQ was more bioavailable, as denoted by higher serum concentrations in both normal and infected mice where elevated K_a_, AUC_0–24_, C_max_, and t_1/2e_ with a decrease in k_el_ were demonstrated. The AUC_0–24_ for SLN-PZQ in normal and *Schistosoma mansoni*-infected groups was almost nine- and eight-fold higher, respectively, than that for M-PZQ in corresponding groups. In normal and *S. mansoni*-infected mice, SLN-PZQ was detectable in serum at 24 h, while M-PZQ completely vanished 8 h post-treatment. Additionally, enhanced absorption with extended residence time was recorded for SLN-PZQ. Compared to M-PZQ, SLN-PZQ revealed superior antischistosomal activity coupled with enhanced bioavailability in all treated groups where higher percentages of worm reduction were recorded with all dosages tested. This effect was especially evident at the lower dose levels. The ED_95_ of SLN-PZQ was 5.29-fold lower than that of M-PZQ, with a significantly higher reduction in both the hepatic and intestinal tissue egg loads of all treated groups and almost complete disappearance of immature deposited eggs (clearly evident at the low dose levels).

**Conclusions:**

SLN-PZQ demonstrated enhanced PZQ bioavailability and antischistosomal efficacy with a safe profile despite the prolonged residence in the systemic circulation.

## Background

Schistosomiasis, a neglected tropical parasitic disease, is endemic in more than 75 countries [1]. Over 200 million individuals are infected worldwide, and schistosomiasis is within the top ten diseases for years lived with disability in sub-Saharan countries [2]. Despite this considerable disease burden, at present, there is no effective vaccine, the intermediate host is not always easily defeated and chemotherapy remains the primary approach for disease control. Praziquantel (PZQ) remains the only effective frontline drug to treat the parasite and is currently characterized by its exclusive and extensive use as an important antischistosomal drug [3]. Although mass administration campaigns of PZQ as well as preventive measures through chemotherapy have shown a significant impact on schistosomiasis, the efficacy concerns are debatable in addition to the possible development of drug resistance [4–7]. Since PZQ does not show a 100% cure rate [8, 9], the surviving worms might develop resistance quickly; hence, this resistance might be passed on to the next generations of *Schistosoma* [10, 11]. The failure to achieve complete cures in PZQ-treated populations may be linked to the insufficient long-lasting effect of PZQ and its low bioavailability, which is attributed to the hydrophobic nature of the drug [11, 12]. PZQ is a class II compound (high permeability, low solubility) and is highly lipophilic. The drug crosses the gastrointestinal tract by passive diffusion to quickly reach the bloodstream and undergoes rapid metabolism [13, 14]. This rapid metabolism might account for the insusceptibility of juvenile and immature worms to PZQ in the systemic circulation and might raise concerns about the long-term impact of mass treatment campaigns. Larger doses or repetitive treatments were recommended for enhancing treatment efficacy [15], although these treatments might account for the higher frequency of side effects [16]. In a meta-analysis involving 273 clinical studies, the series of side effects reported were related to the recommended higher dose of PZQ [9] and may also be the main driver for the evolution of *Schistosoma* resistance to treatment. Moreover, repetitive treatments definitely represent a financial burden on developing countries, justifying the search for alternatives as well as the development of methods to overcome existing drug drawbacks.

PZQ in nanoformulation may overcome its known drawbacks (quick first pass metabolism in the liver) with its consequent reduced efficacy, specifically against the immature forms in the systemic circulation. Several promising drug delivery systems were examined for PZQ [17–20]. Incorporating drugs into nanocarriers, specifically “solid lipid nanoparticles (SLNs)”, has a superior advantage over common nanoformulations with respect to drug targeting, release, long-shelf stability, low toxicity, better bioavailability and compatibility with several routes of administration [21, 22]. Moreover, SLNs could resolve drawbacks, including undesirable taste, possible irritation and gastrointestinal adverse reactions with high safety margins and high biocompatibility over other conventional nanoformulations [23].

In the present study, simple, cost-effective formulations of PZQ in solid lipid nanoparticles (SLN-PZQ) were tested in experimental murine schistosomiasis with the aim of enhancing PZQ solubility, bioavailability and efficacy.

## Methods

### Experimental animals

CD1 male Swiss albino mice, with an average weight of 20 ± 2 g, were bred and maintained at the Schistosome Biology Supply Center (SBSC) of Theodor Bilharz Research Institute (TBRI), Giza, Egypt. All animal experiments were conducted with respect to guidelines for the care and use of laboratory animals of the National Institutes of Health (NIH, 1996) and its amendments.

### Drugs

PZQ [Distocide; Egyptian International Pharmaceuticals Industries Co (EIPICO), Cairo, Egypt] tablets were ground into powder, marked as “market PZQ (M-PZQ)” and given orally as a suspension in 2% Cremophor-El (Sigma-Aldrich, St. Louis, MO, USA). Fresh batches of drug doses were administered by oral gavage using a stainless-steel oral cannula.

### Preparation of SLN-PZQ

Different SLN-PZQ formulations were prepared (Table 1) using the pure form of PZQ (EIPICO). The lipid phase in SLN preparation consisted of variable ratios of plurol oleique (P.O.) to Labrafil, in addition to constant 0.1 mg Lauroglycol 90. These components were melted at 5 °C above the melting point of the lipid used, and PZQ was well dispersed therein to obtain a drug-lipid mixture. The drug-lipid mixture was sonicated for 30 min (Elmasonic S 30 (H);

**Table 1:** Composition of solid lipid nanoparticle formulations and their physicochemical properties when loaded with PZQ

SLN formula	Composition of SLN formulations			Physicochemical properties			
	Labrafil: Plurol oleique	Lauroglycol 90 (mg)	Labrasol (mg)	Particle size (nm)^a^	PDI	ZP (mV)	EE (%)^a^
F1	1:1	0.1	0.1	285.45 ± 4.39	0.843	− 14.5	5.46 ± 0.23
F2	1:3	0.1	0.1	302.3 ± 5.27	0.657	− 17.3	3.02 ± 0.11
F3	1:5	0.1	0.1	123.5 ± 2.19	0.675	− 18.5	32.04 ± 2.19
F4	1:1	0.1	0.2	140.4 ± 2.49	0.654	− 23.4	31.12 ± 3.02
F5	1:3	0.1	0.2	155.3 ± 3.01	0.765	− 24.6	54.56 ± 4.28
F6	1:5	0.1	0.2	133.6 ± 8.22	0.546	− 26.4	91.26 ± 3.39
F7	1:1	0.1	0.4	105.3 ± 4.39*	0.314	− 35.2	109.21 ± 2.83*
F8	1:3	0.1	0.4	87.32 ± 5.19*	0.432	− 38.3	101.12 ± 3.28*
F9	1:5	0.1	0.4	118.4 ± 7.10*	0.321	− 35.4	100.23 ± 1.46*

*Notes*: Praziquantel-loaded SLNs were composed of plurol oleique (P.O.), Labrafil and Lauroglycol 90 as core matrices Labrasol in 10 ml distilled water was incorporated at concentrations of 0.1, 0.2 or 0.4 mg to each of the three Labrafil:plurol oleique ratios. Physiochemical properties were assessed for each formulation

^a^Values represent the means ± standard deviation (SD)

* Significant differences from the different SLN formulations at *P* < 0.05

*Abbreviations*: SLN, solid lipid nanoparticle; PDI, polydispersity index; ZP, zeta potential; EE, entrapment efficiency

Elma, Singen, Germany). An aqueous phase containing either 0.1, 0.3 and 0.5 mg of Labrasol in 10 ml of distilled water was heated up to the same temperature as the molten lipid phase, and the hot lipid phase was poured onto the hot aqueous phase with homogenization at 25,000× *rpm* for 3 min. PZQ-loaded SLNs were finally obtained by allowing hot nanoemulsions to cool to room temperature. Blank SLNs were prepared using the same variables but without the drug [24]. SLNs were lyophilized using a programmable freeze-dryer (alpha 1–4 LSC; Christ, Osterode am Harz, Germany).

### Characterization of SLN-PZQ

#### Particle size and polydispersity index (PDI)

The size of the prepared SLNs and the PDI were measured after reconstitution with ultra-purified water using a particle size analyser (Zetasizer 2000; Malvern Instruments, Malvern, UK). Particle size measurement ensured the nanosize range of the prepared formulations, while the PDI validated the uniformity of the nanoparticle size distribution [25].

#### Zeta potential

As a quantitative guide to particle stability, the zeta potential of SLNs was determined by measuring electrophoretic mobility using a zeta sizer (Zetasizer 2000). Prior to the electrophoretic mobility measurements, all the samples were diluted with ultra-purified water, and the measurements were carried out at 25 °C. The conversion of electrophoretic mobility to the zeta potential was performed using the following Helmholtz–Smoluchowski equation [26]: ζ = E (4πη/ε) where ζ is the zeta potential (mV), E is the electrophoretic mobility, η is the viscosity of the dispersion medium (water 0.8904 cp) and ε is the dielectric constant of the solvent (water, 78.54).

#### Entrapment efficiency (EE)

The prepared nanoparticle dispersion was centrifuged at 14,000× *rpm* for 1 h at 0 °C using a cooling centrifuge (Sigma-3k30; Sigma-Aldrich, Munich, Germany). The supernatant was then analysed for the free drug content. The concentration of PZQ in the supernatant was determined by UV-visible spectrophotometry at 263.8 nm. Entrapment efficiency (EE) was calculated using the following equation: EE (%) = (PZQ initial–PZQ supernatant) / PZQ initial × 100.

### *In vitro* release studies

To assess the percentage of drug released during the transit time in the stomach, we examined the *in vitro* release of formulations exhibiting optimum properties regarding physiochemical characteristics. The *in vitro* release of PZQ-loaded SLNs was examined in 900 ml of 0.1 N HCl, and the temperature was maintained at 37 °C with a paddle operated at 50× *rpm*. The formulations were secured by clamps in a non-rate controlling dialysis bags. An aliquot of 5 ml was withdrawn at pre-determined intervals of 0.25, 0.5, 1.5 and 2 h and then substituted with 5 ml of 0.1 N HCl. This process was performed using a USP dissolution tester (apparatus II, model DT-D; Erweka Apparatebau GmbH, Heusenstamm, Germany). Aliquots were analysed spectrophotometrically at 263.8 nm.

### Pharmacokinetic studies

The formulation of optimum properties regarding physicochemical characteristics and *in vitro* release was selected for pharmacokinetic studies. Two batches comprising 220 normal mice were used for the assessment of pharmacokinetics for “M-PZQ” and “SLN-PZQ” after their administration in a single oral dose of 500 mg/kg. Each batch was subdivided into 11 groups of 10 mice each according to time of sacrifice (by decapitation), i.e. 2, 5, 15, 30 min and 1, 2, 4, 6, 8, 14 and 24 h after drug administration. Two other batches of *S. mansoni-*infected mice, matching the number of normal animals, followed the same procedure. At times of sacrifice, blood samples were collected, and sera were separated and stored at − 70 °C until assayed. Extraction and estimation of PZQ in the different serum samples were performed using high-performance liquid chromatography (HPLC) on a series 3000 Dionex Ultimate (Dionex, Amsterdam, The Netherlands) adhering to a modification of the method described by Xiao et al. [27]. The column flow rate was 2 ml/min, the mobile phase used was 42% acetonitrile/H_2_O and the detector wavelength was set at 210 nm. For PZQ concentration estimation, 0.5 ml of sera was extracted with 3-ml aliquots of ethyl acetate and centrifuged at 1850× *g* for 15 min. Two-milliliter aliquots of the extracts were evaporated to dryness at 25 °C under a nitrogen stream, and the residue was then re-suspended in 200 μl of acetonitrile and shaken on a vortex mixer for 1 min. The sample (25 µl) was then injected into a Nova Pak C18, 60 Å, 4 μm, 3.9 × 150 mm HPLC column (Millipore Co., Milford, MA, USA) equipped with a series 200 programmable multi-wavelength detector (Perkin Elmer; Norwalk, CT, USA). The calibration curve was linear between 0 and 3.2 μg/ml (correlation coefficient (*r*) = 0.9984). The coefficients of variation for within- and inter-day reproducibility were 1–4% depending on the drug concentration.

Pharm PCS software v.4.2 was used for computing pharmacokinetic parameters based on the classic method of residuals and the least square technique for curve fitting [28]. Coefficients and exponents of the fitted function were used to calculate the theoretical maximum concentration (C_max_) and the time to maximum concentration (T_max_) of PZQ. The corresponding elimination half-lives (t_1/2e_) were calculated as ln 2/elimination rate constant (k_el_). The area under the serum concentration-time curve (AUC_0–24h_) was calculated by applying the linear trapezoidal method.

### Antischistosomal efficacy

#### Schistosoma mansoni infection of animals

*Schistosoma mansoni* cercariae, obtained from laboratory-bred infected *Biomphalaria alexandrina* snails, were used to infect mice. The body immersion technique was used for the infection with 80 ± 10 cercariae per mouse [29].

#### Animal groups

*Schistosoma mansoni*-infected mice were randomly allocated into two batches. Seven weeks post-infection, the animals of each batch were subdivided into six groups of 6–8 mice each. Five of these groups received either M-PZQ or SLN-PZQ in total oral doses of 62.5, 125, 250, 500 or 1000 mg/kg divided equally on 5 consecutive days, while the sixth group was left untreated as the respective control.

#### Recovery of S. mansoni worms and estimation of PZQ ED_95_

Two weeks after the end of treatment, mice were sacrificed by decapitation, and the hepatic and portomesenteric vessels were perfused to recover and count worms, taking care to separate hepatic from portomesenteric worms [30]. The percent worm reduction was calculated to estimate the PZQ 95% effective dose (ED_95_) [5]. The percent reduction in worm burden in each treated group was calculated using the following equation: No. of worms in the control group − No. of worms in the treated group/No. of worms in the control group × 100. The ED_95_ (effective dose required to kill 95% of adult worms) was calculated using Probit analysis of a plot of worm reduction percentages *versus* amounts of drug administered using GraphPad Prism software v.6.0.

#### Determination of tissue egg load and oogram profile (percentage of eggs in different developmental stages)

Portions of the liver and intestine were examined to determine the number of eggs per gram of liver and intestine tissue [31]. Another portion of the small intestine was used to determine the percentage of egg developmental stages. A dose of a drug was considered to have a definite activity against *S. mansoni* when the oogram revealed 50% or more mature eggs and the absence of one or more stages of immature eggs [32].

### Statistical analysis

The results are expressed as the mean ± SEM. One-way analysis of variance (ANOVA) followed by Tukey’s *post-hoc* test was performed using GraphPad Prism software to detect the significance of differences between the means of different groups. The results were considered significant if *P* < 0.05.

## Results

### Preparation of SLN-PZQ

PZQ-loaded SLN dispersions were composed of P.O., Labrafil and Lauroglycol 90 as core matrices. Despite the liquid nature of the used lipids, formulations showed an emulsion appearance, which was attributed to the polyoxyl nature of Labrafil that can form hydrogen bonds with water, resulting in a network-like structure capable of swelling (Table 1).

### Characterization of SLN-PZQ

#### Particle size and polydispersity index (PDI)

The particle sizes of all prepared SLNs ranged between 87.32–302.3 nm. No general trend was observed for the increase or decrease in particle size with increasing Labrafil:P.O. ratios in each of the studied Labrasol concentrations. However, increasing the concentration of Labrasol from 0.1 mg to 0.4 mg shifted the average particle size to significantly smaller values (ANOVA, *F*_(3,27)_ = 9.34, *P* = 0.023). The polydispersity index is a ratio that provides information about the homogeneity of particle size distribution in a given system. Generally, the prepared formulations showed high PDIs except for formulas F7 and F9 where narrow dispersity was recorded (Table 1).

#### Zeta potential

Zeta potential is an indicative of probable physical stability of a formulation. Nanoparticles with zeta potential values more than 25 mV showed high degrees of stability [33]. Formulas with the highest concentration of Labrasol (F7, F8 and F9) showed significantly higher zeta potential (ANOVA, *F*_(3,27)_ = 8.57, *P* = 0.026) than did other formulations (Table 1). Due to the negative charge of the lipid phase, the zeta potential of all formulations was negative.

#### Entrapment efficiency (EE)

Entrapment efficiency is used to assess the capability of the prepared SLNs to efficiently entrap PZQ. All SLN formulations prepared with a higher concentration of surfactant showed significantly higher EE (F7: 109.2%; F8: 101.12%; and F9: 100.23%). Additionally, increasing the particle size above the average range (F1: 285.45 nm and F2: 302.3 nm) drastically decreased the EE (Table 1).

### *In vitro* release studies

**Fig. 1:**
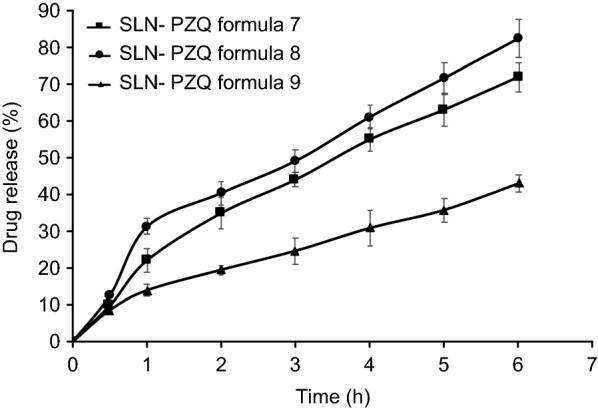
In vitro percentage PZQ released over time from solid lipid nanoparticle-PZQ formulations. In vitro release studies were performed to select SLN formulations with optimum physiochemical properties (Formulas 7, 8 and 9). This selection was performed using a USP dissolution tester, and aliquots were analysed spectrophotometrically. Values are presented as the means ± SD. Abbreviation: SLN-PZQ, praziquantel-loaded solid lipid nanoparticle

Formulas F6, F7 and F8 exhibited optimum properties regarding particle size, drug entrapment and physical stability and were subjected to *in vitro* release studies. *In vitro* release, expressed by transit time in the stomach, revealed that F9 possessed the largest particle size (118.4 nm) and showed the lowest percentage of drug release over 6 h, while F8 with a smaller diameter (87.32 nm) showed a higher percentage of drug release (Fig. 1). F8 showed 31.18% burst release at 1 h, whereas F7 and F9 did not express the same profile. F7 showed the best physicochemical characteristics and was selected for the *in vivo* biological investigation involving bioavailability and efficacy studies.

### Pharmacokinetic studies of SLN-PZQ and M-PZQ in normal and *S. mansoni*-infected mice

**Fig. 2:**
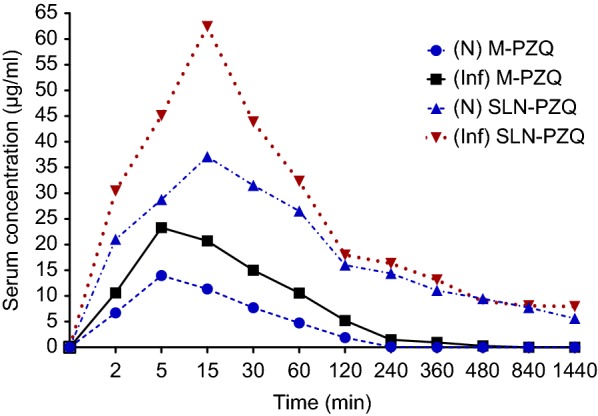
Serum concentration-time curves of SLN-PZQ and or market PZQ at a single dose of 500 mg/kg. Abbreviations: (N) M-PZQ, serum concentration of market praziquantel in normal mice; (Inf) M-PZQ, serum concentration of market praziquantel in *S. mansoni*-infected mice; (N) SLN-PZQ, serum concentration of solid lipid nanoparticle praziquantel in normal mice; (Inf) SLN-PZQ, serum concentration of solid lipid nanoparticle praziquantel in *S. mansoni*-infected mice

Compared to M-PZQ, SLN-PZQ showed higher PZQ concentrations in the sera of both normal and *S. mansoni*-infected mice, as demonstrated by the elevated absorption rate constant (K_a_), AUC_0–24_, C_max_, and t_1/2e_ with a decrease in k_el_ (Table 2). The values obtained for AUC_0–24_ for the SLN-PZQ group were approximately 9-fold and 8-fold higher than those recorded for the M-PZQ group in both normal and *S. mansoni*-infected mice, respectively. Compared to mice treated with M-PZQ, normal and *S. mansoni*-infected mice treated with SLN-PZQ showed a significant increase in K_a_, C_max_ and t_1/2e_ by 2.14- and 1.99-fold; 2.33- and 2.64-fold; and 6.41 and 6.17-fold, respectively, while K_el_ significantly decreased by 5.77- and 6.30-fold, respectively (Table 2). The serum concentration of SLN-PZQ was detectable at 24 h, while that of M-PZQ vanished 8 h post-treatment in both normal and *S. mansoni*-infected mice (Fig. 2).

**Table 2:** Pharmacokinetic parameters of SLN-PZQ vs market PZQ at a single dose of 500 mg/kg

Animal group	Treatment	Pharmacokinetic parameters (mean ± SEM)					
		k_a_ (h^−1^)	k_el_ (h^−1^)	t_1/2e_ (h^−1^)	AUC (μg h/ml)	C_max_ (μg/ml)	T_max_ (h)
Normal	M-PZQ	6.52 ± 0.05	1.27 ± 0.01	0.55 ± 0.00	14.03 ± 0.98	15.98 ± 1.46	0.14 ± 0.04
	SLN-PZQ	13.98 ± 1.30*	0.22 ± 0.03*	3.53 ± 0.60*	123.75 ± 11.39*	37.24 ± 2.07*	0.29 ± 0.04
*S. mansoni-*infected	M-PZQ	11.23 ± 0.86^$^	0.63 ± 0.01^†^	1.11 ± 0.02^†^	34.61 ± 2.50^†^	24.31 ± 1.19	0.14 ± 0.04
	SLN-PZQ	22.34 ± 0.67^#†^	0.10 ± 0.01^#†^	6.85 ± 0.60^#†^	286.33 ± 13.54^#†^	64.29 ± 7.78^#†^	0.22 ± 0.03

* Significant differences between SLN-PZQ and M-PZQ in normal treated mice at *P* < 0.05

^#^Significant differences between SLN-PZQ and M-PZQ in *S. mansoni*-infected treated mice at *P* < 0.05

^†^Significant differences of SLN-PZQ or M-PZQ in *S*. *mansoni*-infected mice compared to respective normal mice at *P* < 0.05

*Abbreviations*: k_a_, absorption rate constant; k_el_, elimination rate constant; t_1/2e_, half-life of elimination; AUC, area under concentration-time curve; C_max_, maximum concentration; T_max_, time to reach C_max_; SLN-PZQ, solid lipid nanoparticle praziquantel; M-PZQ, market praziquantel

### Antischistosomal efficacy

**Fig. 3:**
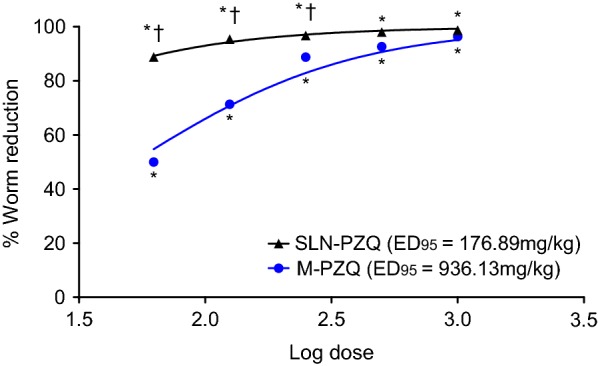
Computer-adjusted drug dose-response curves for M-PZQ and SLN-PZQ. The curves were fitted to results calculated using GraphPad Prism software v.6.01 and were plotted assuming that all had a minimum of 0% and a maximum of 100%. *Significant worm reduction in S. mansoni-infected mice treated with SLN-PZQ and M-PZQ vs the corresponding infected untreated controls at P < 0.05. ^†^Significant worm reduction in S. mansoni-infected mice treated with SLN-PZQ vs that in mice treated with M-PZQ at P < 0.05. Abbreviations: SLN-PZQ, solid lipid nanoparticle praziquantel; M-PZQ, market praziquantel; ED_95_, effective dose required to kill 95% of adult worms

**Fig. 4:**
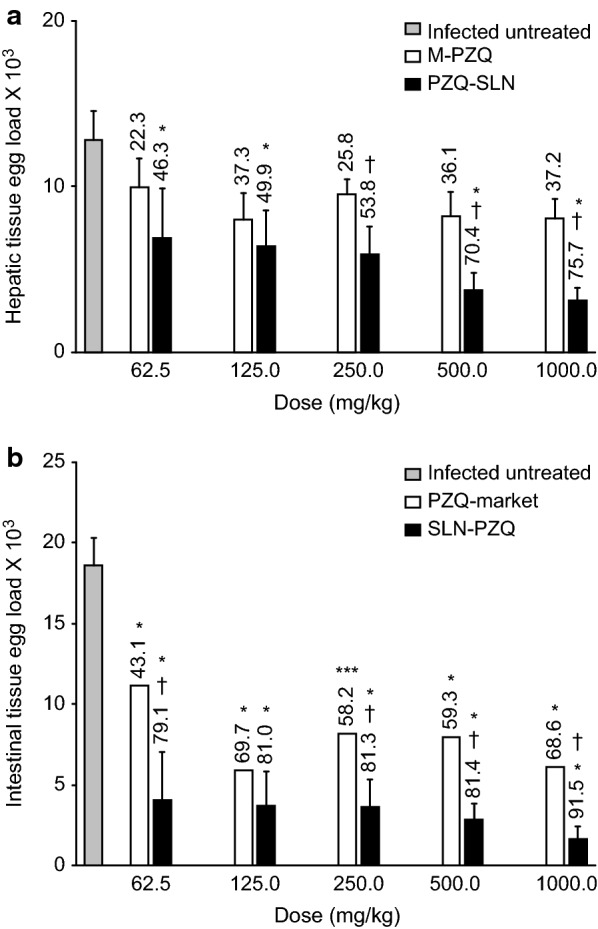
Hepatic (**a**) and intestinal (**b**) tissue egg-load in infected mice treated with M-PZQ or SLN-PZQ. Mice were sacrificed 14 days after treatment. Data are presented as the means ± SEM. n = 6–8 mice per group. Number in parentheses represents the percentages of egg reduction vs infected untreated control. *Significant difference vs infected untreated control at P < 0.05. ^†^Significant difference between SLN-PZQ and M-PZQ treatment at P < 0.05. Abbreviations: SLN-PZQ, solid lipid nanoparticle praziquantel; M-PZQ, market praziquantel

**Fig. 5:**
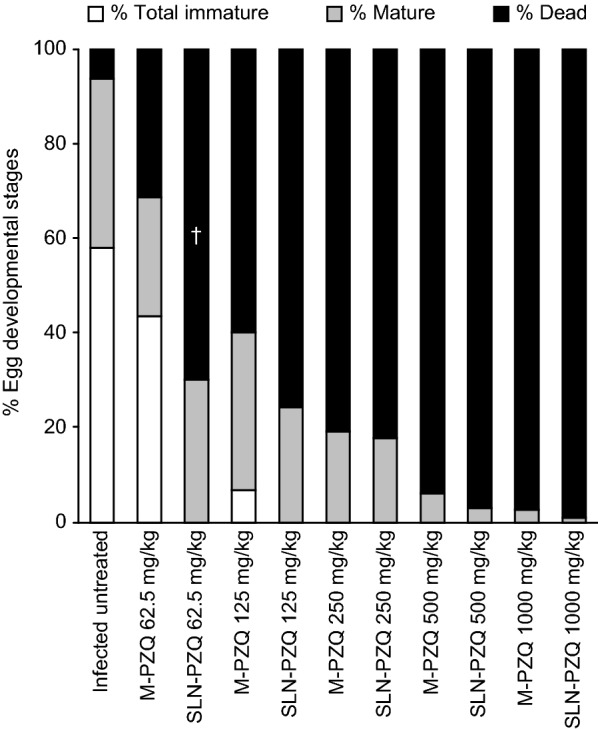
Percentage egg developmental stages (oogram pattern) in infected mice treated with SLN-PZQ or market PZQ. Mice were sacrificed 14 days after treatment. Data are presented as the means ± SEM. n = 6–8 mice per group. ^†^Significant difference vs M-PZQ at P < 0.05. Abbreviations: SLN-PZQ, solid lipid nanoparticle praziquantel; M-PZQ, market praziquantel

At 14 days post-treatment, the percent total worm reduction was higher in *S. mansoni*-infected mice treated with rising doses of SLN-PZQ than in mice treated with M-PZQ (Table 3), but the differences (86.5, 94.4 and 96.1% for SLN-PZQ *vs* 51.5, 72.2 and 89.1%, for M-PZQ) were significant with the small doses tested (62.5 mg/kg: ANOVA, *F*_(2,15)_ = 18.45, *P* < 0.0001; 125 mg/kg: ANOVA, *F*_(2,15)_ = 8.36, *P* = 0.0036; 250 mg/kg: ANOVA, *F*_(2,15)_ = 6.92, *P* = 0.0074), respectively. The ED_95_ of SLN-PZQ was 5.29-fold lower than that of M-PZQ (176.89 mg/kg *vs* 936.13 mg/kg for M-PZQ) (Fig. 3). Moreover, compared to M-PZQ treatment, SLN-PZQ treatment resulted in a significantly higher reduction in both the hepatic tissue egg load at the higher doses tested and in the intestinal tissue egg load with all doses recorded (Fig. 4a, b). Compared to M-PZQ, all doses of SLN-PZQ resulted in the complete absence of immature eggs in the oogram pattern (Fig. 5). M-PZQ started to show the same finding when the 250 mg/kg dose was tested. Compared to M-PZQ, SLN-PZQ significantly increased the percentage of dead eggs, and the difference was significant (ANOVA, *F*_(2,15)_ = 9.55, *P* = 0.0021) with the lowest dose tested (62.5 mg/kg). Although no significant differences were observed at the higher doses of SLN-PZQ tested, an apparently higher reduction in the percentages of total mature eggs was recorded after SLN-PZQ treatment than after M-PZQ treatment (Fig. 5).

**Table 3:** Worm distribution in the hepatic and portomesenteric vessels of S. mansoni-infected treated mice

Dose (mg/kg)	Animal group	Total no. of worms	Total no. of male worms	Total no. of female worms	Total no. of hepatic worms	Total no. of portomesenteric worms
Control		21.00 ± 0.59	12.44 ± 1.6	8.56 ± 1.0	5.33 ± 1.14	15.66 ± 1.98
62.5	M-PZQ	10.17 ± 0.4 (51.59)*	6.33 ± 1.0 (49.09)*	3.83 ± 0.7 (55.22)*	4.66 ± 0.8	5.5 ± 0.62*
	SLN-PZQ	2.83 ± 0.7 (86.51)*^†^	2.67 ± 0.7 (78.56)*^†^	0.17 ± 0.1 (98.05)*^†^	1.00 ± 0.26*^†^	1.83 ± 0.60*^†^
125	M-PZQ	5.83 ± 0.7 (72.22)*	3.83 ± 1.0 (69.19)*	1.33 ± 0.2 (84.42)*	2.83 ± 0.98*	2.33 ± 0.76*
	SLN-PZQ	1.17 ± 0.4 (94.44)*^†^	0.83 ± 0.3 (92.50)*^†^	0.33 ± 0.2 (95.33)*^†^	0.33 ± 0.21*^$^	0.83 ± 0.98*
250	M-PZQ	2.29 ± 0.1 (89.10)*	2.0 ± 0.2 (83.92)*	0.29 ± 0.1 (96.66)*	0.57 ± 0.4*	0.57 ± 0.20*
	SLN-PZQ	0.83 ± 0.3 (96.05)*^†^	0.67 ± 0.2 (94.64)*	0.17 ± 0.1 (98.05)*	0.33 ± 0.5*	0.33 ± 0.20*
500	M-PZQ	1.43 ± 0.2 (93.19)*	0.86 ± 0.3 (93.11)*	0.57 ± 0.2 (93.32)*	1.17 ± 0.38*	0.29 ± 0.49*
	SLN-PZQ	0.50 ± 0.3 (97.62)*	0.33 ± 0.3 (97.32)*	0.33 ± 0.2 (98.05)*	0.5 ± 0.34*	0.00 ± 0.00*
1000	M-PZQ	0.75 ± 0.3 (96.43)*	0.63 ± 0.3 (94.98)*	0.13 ± 0.1 (98.54)*	0.75 ± 0.31*	0.00 ± 0.00*
	SLN-PZQ	0.33 ± 0.2 (98.43)*	0.33 ± 0.2 (97.32)*	0.00 ± 0.0 (100.0)*	0.17 ± 0.16*	0.16 ± 0.16*

*Notes*: Data are presented as the means ± SEM. *n* = 6–8 mice per group. Mice were sacrificed 14 days after treatment. Number in parentheses represents the percentage worm reduction *versus* the infected untreated control. Percentage of worm reduction = (mean number of worms in control group - mean number of worms in treated group)/mean number in control group × 100

* Significant differences of SLN-PZQ or M-PZQ *versus* parallel infected untreated control at *P* < 0.05

^†^Significant differences of SLN-PZQ *versus* parallel values of M-PZQ at *P* < 0.05

*Abbreviations*: SLN-PZQ, solid lipid nanoparticle praziquantel; M-PZQ, market praziquantel

## Discussion

The search for innovative drugs is paramount in medicine, but the challenge is greater for neglected tropical diseases where little is invested in developing such treatments. The pipeline is almost dry for tropical diseases of the poor due to the marginal or almost non-existent profits and the needs are unmet for these medical conditions [34] where the costs for the discovery and development are too high to achieve therapeutic excellence and capture market economies [35].

This work focused on improving the biological characteristics of the existing drug to treat schistosomiasis, PZQ. Working on existing drugs simplifies and shortens the long and difficult process of drug discovery. On average, it takes at least ten years for a new medicine to complete the journey from initial discovery to the marketplace [36]. Although PZQ is endowed with excellent properties, e.g. effective against the main schistosomes pathogenic for humans with almost minimal side effects [37], drug efficacy is a concern because a 100% cure rate has not been achieved. Additionally, there is fear of worms surviving this treatment and potentially passing to the next generation leading to development of resistance. In addition, the drug’s poor solubility in water and consequently low absorption restricts its delivery *via* the oral route [38, 39]. PZQ is rapidly metabolized with less efficacy against immature worms, and large drug doses are required to achieve a sufficient concentration in larval tissue. In the present study, to overcome the drug drawbacks, we used SLNs were used as a delivery system for PZQ. To assess the bioavailability and efficacy of SLN-PZQ, we examined the bioavailability of the new formulation *in vivo* in normal and S. *mansoni*-infected mice, while the efficacy of SLN-PZQ was studied *in vivo* using *S. mansoni*-infected mice and compared to that of conventional market PZQ.

SLNs as a delivery system have the advantage of increasing the surface area and quantum effects, thus enhancing the physicochemical characteristics, involving reactivity, strength, electrical characteristics and *in vitro* behaviour. Nine PZQ delivery systems of different SLN compositions (Labrafil/Lauroglycol) were primarily prepared to select the most promising formula with respect to physiochemical characteristics. Assessing the physicochemical characteristics of particle size, drug release, zeta potential and EE revealed that formula 7 with medium-sized particles, high EE, high stability, and particle uniformity is expected to show gradual, persistent drug release [40, 41]. This SLN-PZQ formulation showed a particle size of 105 nm, which is smaller than other particles used in the *in vitro* studies of PZQ-loaded solid lipid nanoparticles against *S. mansoni* [42]. Particle sizes smaller than 400 nm reportedly improve the bioavailability of drugs [43]. The high concentrations of non-ionic surfactant (Labrasol) through its steric properties enhanced the stability [44] of these nanosized particles, as shown by the higher zeta potential values for SLN-PZQ formula 7. The surfactant used lowers the surface tension at the interface of the particles [45], causing partitioning of particles, hence resulting the formation of stabilized nanoparticles with smaller size and larger surface area. Notably, although formula 8 showed a higher drug release profile, the 31.18% burst release at 1 h in addition to the higher PDI affected its stability profile [46].

In the present study, pharmacokinetic assessment of both M-PZQ and SLN-PZQ in normal and *S. mansoni*-infected mice showed enhanced PZQ bioavailability of both M-PZQ and SLN-PZQ in the *S. mansoni*-infected groups. These results are in agreement with previous experimental studies in mice [17, 18, 47, 48] as well as in human patients infected with *S. mansoni* [49, 50]. This enhancement might be related to the inflammatory reaction as a result of egg deposition [51], causing inhibition of cytochrome P450 (CYP450) and CYP450 reductase activities that were recorded in mice and human patients infected with *S. mansoni* [52, 53]. SLN-PZQ showed more absorption, bioavailability and higher serum concentrations than did M-PZQ, as denoted by elevated K_a_, AUC_0–24_, C_max_, and t_1/2e_ with a decrease in k_el_. The AUC_0–24_ for the SLN-PZQ groups were almost 9- and 8-fold higher than those for M-PZQ in corresponding groups of normal and infected mice, respectively. In addition, SLN-PZQ showed increases in C_max_ and t_1/2e_ by 2.33- and 2.61- and 6.4- and 6.17-fold while k_el_ decreased by 5.77- and 6.30-fold in corresponding groups of normal and infected mice, respectively. Improved dissolution of PZQ when administered in the SLN formulation was demonstrated in this work where the K_a_ was increased by 2.14- and 1.99-fold compared with that in the M-PZQ formulation in corresponding groups of normal and infected mice, respectively. The improved dissolution of PZQ recorded is expected to enhance drug absorption as well as increase the residence period of the drug in the systemic circulation, hence prolonging the exposure of the parasite to the drug. The serum concentration of SLN-PZQ was detectable (7.96 μg/ml) in *S. mansoni*-infected mice 24 h after administration, while M-PZQ completely disappeared 8 h post-treatment. Additionally, a remarkable extended release pattern of SLN-PZQ has been reported and related to its enhanced bioadhesive nature to the wall of the gastrointestinal tract while benefiting from lipid protection in less exposure to enzymatic degradation [41]. This finding was revealed by a 6.4-fold increase in the serum t_1/2e_ of SLN-PZQ compared to that of M-PZQ in normal and infected mice. The strikingly increased C_max_ following the administration of SLN-PZQ is consistent with previous studies showing elevated plasma concentrations of SLN-PZQ [41, 54]. The elevated C_max_ is also related to the possible high PZQ eruption release from the outer shell of SLNs at the initial phase [41]. By contrast, when nanoparticles were loaded in poly methyl methacrylate (PMMA), a 3-fold lower C_max_ value of PZQ nanoparticles was reported compared to that of conventional PZQ nanoparticles. This result was attributed to the non-bioadhesive nature of PMMA and the incomplete release of the drug from the polymer matrix [19]. The particle surface properties in addition to the uniformity and proximity of the drug-loaded nanoparticles to the absorptive region of the gastrointestinal tract seem to govern the residence time, hence decreasing the time the drug remained in plasma, resulting in a low plasma concentration, as in the case of PMMA [55]. In this respect, it is important to highlight that the different preparation techniques together with the characterization of physical and chemical properties of the material used as a matrix for nanoparticle systems could interfere with nanoparticle drug absorption [56].

In the present study, the pharmacokinetic results revealed a delayed release effect of SLN-PZQ over M-PZQ in both normal and infected mice. These findings are aligned with studies revealing a significant increase in T_max_ of the SLN suspension of PZQ [41] and contradicting other studies that showed comparable T_max_ values of PZQ nanoparticles and conventional PZQ [19].

In this work, it was essential to examine the biological activities of this new formulation and whether this enhanced bioavailability results in higher efficacy in a model of murine schistosomiasis. *In vivo* studies of the efficacy of SLN-PZQ in schistosomiasis are limited, and the conducted *in vitro* studies focused on examining the possible enhanced physicochemical characteristics [41, 42]. With respect to other parasitic infections, SLN formulations have shown promising findings over conventional PZQ in cestodal infection with the use of a low dose (5 mg/kg) in dogs infected with *Echinococcus granulosus* [54]. Additionally, using nanoprecipitation, a PZQ nanosuspension showed better efficacy against the cysticerci of the cestode parasite *Taenia crassiceps* [20]. In the present study, the enhanced bioavailability of PZQ in the SLN delivery system was recorded in addition to higher efficacy in mice infected with *S. mansoni* and treated with this formulation. Parasitological findings revealed an increase in the percentage of worm reduction by 1.67-, 1.30- and 1.06-fold in infected mice treated with SLN-PZQ at doses of 62.5, 125 and 250 mg/kg, respectively. Moreover, the ED_95_ was reduced to 176.89 mg/kg for SLN-PZQ *vs* 936.13 mg/kg for M-PZQ. This enhanced efficacy is a result of the higher drug concentration in serum and decreased elimination as well as the prolonged time in the circulation. The antischistosomal effect of PZQ is associated with the absolute height of the maximal plasma concentration as well as the length of exposure to the drug [57]. Previous studies on nanoclay formulation of PZQ revealed enhanced bioavailability and higher efficacy in *S. mansoni*-infected mice, with a significant reduction in the dose by almost three times the conventional PZQ dose to achieve 50% killing of *S. mansoni* worms [18]. Moreover, compared to conventional PZQ, the polymeric formulation of PZQ in solid dispersion reduced the ED_50_ by twofold [17].

Compared to M-PZQ, SLN-PZQ exhibited a stronger effect on the more troublesome female worms that was more evident at the lower dose levels (Table 3). This finding is in agreement with previous studies that showed superior activity of liposomal-PZQ and PZQ-solid dispersion against female worms [58–60] and the general preferential efficacy of PZQ against female adult worms [61]. In schistosomiasis, tolerance varies among *Schistosoma* species and varies among male and female worms. Male worms have more tolerance for PZQ activity [29, 62, 63].

Compared to untreated control and equivalent doses of M-PZQ, SLN-PZQ at doses of 250, 500 and 1000 mg/kg interrupted worm oviposition capacity, which was reflected as a significant reduction in both the hepatic and intestinal tissue egg load. The lower reduction in the hepatic tissue egg load with certain doses could be related to the higher density of eggs in hepatic tissues compared to intestinal tissue. The tendency of oviposition in the liver tissues after ceasing in the intestinal tissues was recorded upon treatment of *S. mansoni*-infected mice with subcurative doses of PZQ [61]. In the present study, the reduction in tissue egg load in mice treated with SLN-PZQ coupled with complete disappearance of immature eggs, marked reduction in mature eggs and increase in dead eggs was recorded. The complete absence of total immature eggs in the oogram pattern is considered a significant indication of drug efficacy [64] and was especially evident in this work at the lowest doses tested (62.5 mg/kg). M-PZQ showed a similar effect against immature eggs only when the dose was quadrupled to 250 mg/kg. Previous empirical evidence showed that the recommended therapeutic dose of M-PZQ is not effective against immature eggs [65] and that repeating the dose nine days after the initial treatment would insult immature eggs [66].

SLN has superior advantages over conventional nanoformulations in terms of drug targeting, drug release modulation, long-shelf stability, low toxicity, better bioavailability and compatibility with several routes of administration [21, 22]. Several studies revealed an enhanced safety profile for lipid-carrier nanoformulations [23]. Moreover, PZQ showed an enhanced safety profile when incorporated in nanostructured lipid carriers [67], but liposomes and lipid emulsions as drug delivery systems revealed major drawbacks of drug leakage, hydrolysis, possible particle growth and instability during storage [22]. PZQ in nanoclay revealed significantly higher bioavailability and efficacy were recorded; nonetheless, there have been significant changes in haematological analysis and alterations in the cellular electrolyte balance in normal rats following the administration of large doses of montmorillonite clay [68–70]. Although the safety of SLN-PZQ was not tested in the present work, the mouse mortality rate has always been considered a safety indicator.

## Conclusions

SLN potentiated the antischistosomal effect of PZQ where it contributed to a remarkably extended drug release as a result of the protection of the encapsulated drug from enzymatic degradation. Compared to administration of the conventional drug, oral administration of this SLN-PZQ showed significant enhancement of bioavailability and efficacy with a safer profile despite the longer residence in the systemic circulation. Further research involving safety is planned for future studies.

## Data Availability

The data supporting the conclusions of this article are provided within the article.

## Abbreviations

PZQ: praziquantel
SLN: solid lipid nanoparticle
PDI: polydispersity index
EE: entrapment efficiency
ZP: zeta potential
